# The Relevance of Intraoperative Clinical and Accelerometric Measurements for Thalamotomy Outcome

**DOI:** 10.3390/jcm12185887

**Published:** 2023-09-10

**Authors:** Annemarie Smid, D. L. Marinus Oterdoom, Rik W. J. Pauwels, Katalin Tamasi, Jan Willem J. Elting, Anthony R. Absalom, Teus van Laar, J. Marc C. van Dijk, Gea Drost

**Affiliations:** 1 Department of Neurosurgery, University Medical Center Groningen, University of Groningen, Hanzeplein 1, 9713 GZ Groningen, The Netherlands; d.l.m.oterdoom@umcg.nl (D.L.M.O.); r.w.j.pauwels@umcg.nl (R.W.J.P.); k.tamasi@umcg.nl (K.T.); j.m.c.van.dijk@umcg.nl (J.M.C.v.D.); g.drost@umcg.nl (G.D.); 2 Department of Epidemiology, University Medical Center Groningen, University of Groningen, Hanzeplein 1, 9713 GZ Groningen, The Netherlands; 3 Department of Neurology, University Medical Center Groningen, University of Groningen, Hanzeplein 1, 9713 GZ Groningen, The Netherlands; j.w.j.elting@umcg.nl (J.W.J.E.); t.van.laar@umcg.nl (T.v.L.); 4 Department of Anesthesiology, University Medical Center Groningen, University of Groningen, Hanzeplein 1, 9713 GZ Groningen, The Netherlands; a.r.absalom@umcg.nl

**Keywords:** thalamotomy, VIM, insertion effect, microlesion effect, intraoperative neurophysiological neuromonitoring, MDS-UPDRS, accelerometry, Parkinson’s Disease, essential tremor, Holmes tremor

## Abstract

Thalamotomy alleviates medication-refractory tremors in patients with movement disorders such as Parkinson’s Disease (PD), Essential tremor (ET), and Holmes tremor (HT). However, limited data are available on tremor intensity during different thalamotomy stages. Also, the predictive value of the intraoperative tremor status for treatment outcomes remains unclear. Therefore, we aimed to quantify tremor status during thalamotomy and postoperatively. Data were gathered between January 2020 and June 2023 during consecutive unilateral thalamotomy procedures in patients with PD (*n* = 13), ET (*n* = 8), and HT (*n* = 3). MDS-UPDRS scores and tri-axial accelerometry data were obtained during rest, postural, and intention tremor tests. Measurements were performed intraoperatively (1) before lesioning-probe insertion, (2) directly after lesioning-probe insertion, (3) during coagulation, (4) directly after coagulation, and (5) 4–6 months post-surgery. Accelerometric data were recorded continuously during the coagulation process. Outcome measures included MDS-UPDRS tremor scores and accelerometric parameters (peak frequency, tremor amplitude, and area under the curve of power (AUCP)). Tremor intensity was assessed for the insertion effect (1–2), during coagulation (3), post-coagulation effect (1–4), and postoperative effect (1–5). Following insertion and coagulation, tremor intensity improved significantly compared to baseline (*p* < 0.001). The insertion effect clearly correlated with the postoperative effect (*ρ* = 0.863, *p* < 0.001). Both tremor amplitude and AUCP declined gradually during coagulation. Peak frequency did not change significantly intraoperatively. In conclusion, the study data show that both the intraoperative insertion effect and the post-coagulation effect are good predictors for thalamotomy outcomes.

## 1. Introduction

The most common movement disorders with disabling tremors are Parkinson’s Disease (PD), Essential tremor (ET), and Holmes tremor (HT) [1,2]. Tremor in PD is generally observed in the extremities at rest (4–7 Hz) [3,4,5,6]. ET is a monosymptomatic disease, predominated by bilateral action tremor (4–7 Hz) [2,6,7]. Patients with HT suffer from rest, postural, and intention tremors due to low-frequency proximal and distal muscle contraction (<5 Hz) [2,8].

If disabling tremor cannot be suppressed with medication, then a thalamotomy involving a radiofrequency (RF) ablation of the thalamic ventral intermediate nucleus (VIM) is an effective treatment option [9,10,11,12,13,14,15,16,17,18,19,20,21]. Thalamotomies are generally performed unilaterally since bilateral procedures have increased the risk of speech and balance impairments [22,23]. Surgery is generally performed under local anesthesia in awake conditions in order to judge the intraoperative tremor improvement and to minimize side effects. The ever-changing environment in which newer methods of lesioning or ablating the VIM are performed, such as Magnetic Resonance Guided Focused Ultrasound (MRgFUS) [24,25] and Gamma Knife thalamotomy [26], also poses a challenge for the intraoperative monitoring of tremor. Although there are few thalamotomy-related reports on the relevance of intraoperative tremor measurements, the effects measured intraoperatively are hypothesized to predict thalamotomy outcomes.

After the insertion of an RF-lesioning probe in the target area and before heating or stimulation of the tip, immediate tremor alleviation can be observed during surgery: the insertion effect or microlesion effect [27]. In this article, the term ‘insertion effect’ (IE) is maintained. It is postulated that the occurrence of the IE indicates the accurate placement of the RF-lesioning probe within the VIM, underlining the need to adequately monitor tremor intensity intraoperatively [27,28]. As soon as a permanent lesion is inflicted, tremor suppression is immediately observed [21]. This is called the ‘post-coagulation effect’ (PCE).

There are several methods to monitor tremor intensity [27,28,29]. Clinical scales (e.g., Movement Disorder Society Unified PD Rating Scale [MDS-UPDRS]) are often used to assess surgical results [30,31]. However, although the MDS-UPDRS is a standardized scoring system, the results can vary based on the experience of the rater [31,32,33,34,35]. Sensor-based measurements, like tri-axial accelerometry, allow for objective and continuous quantification of tremor intensity [34,36,37]. Several studies have demonstrated that accelerometry can complement clinical assessment during neurosurgery, aiding clinicians in uniformly assessing tremors [38,39,40,41].

Although studies have focused on clinical results and side effects of thalamotomy, there is little data available on the neurophysiological aspects [21,22,42]. Also, there is no consensus on how the IE and PCE relate to the postoperative effect (PE) of unilateral thalamotomy. Therefore, this study focuses on accelerometry measurements during all surgical stages of RF-thalamotomy, including tremor severity in the postoperative phase. The novelty of this work lies in the quantification of tremor intensity during all surgical stages of thalamotomy based on clinical assessment and accelerometry. The aim of this study is to quantify the clinical changes in tremor during the different stages of thalamotomy using both the MDS-UPDRS and tri-axial accelerometry. Secondly, we aim to investigate the relationship between intraoperative findings and clinical outcomes. Lastly, our aim is to investigate the correlation between clinical assessments and accelerometric outcome measures.

## 2. Materials and Methods

This observational study was conducted at the neurosurgical department of the University Medical Center Groningen (UMCG), the Netherlands. The local medical ethics review committee judged that this study does not fall within the remit of the Dutch ‘Research involving human subjects Act’. This study was performed in compliance with the Helsinki Declaration for research on human beings. All participants provided written informed consent for the use of their data.

### 2.1. Study Participants

Data were gathered between January 2020 and June 2023 from consecutive patients with an indication for unilateral RF-thalamotomy. In patients with PD, diagnosis was established according to the UK Brain Bank criteria [43]. For those with ET and HT, the diagnosis was made by movement disorders neurologists and confirmed with a video-assisted surface-EMG accelerometric tremor registration [6]. The treatment plan was discussed and established in a multidisciplinary meeting before inclusion of this study for all participants.

### 2.2. Surgical Procedure

Participants were withheld tremor-suppressing medication for at least 12 h prior to the thalamotomy. Anesthesia administration, frame placement, and target identification were performed as described by Lange and Kremer et al. [28]. In short, the VIM was accessed by penetrating the brain using a bipolar RF probe (TCB013 RF TC Brain Electrode 2 × 2 mm, Inomed Medizintechnik GmbH, Emmendingen, Germany) via a cranial burr hole. Prior to inserting the bipolar RF electrode, baseline tremor intensity of the contralateral arm (relative to the hemisphere being treated) was evaluated (see Section 2.4). Directly after insertion of the electrode in the target, the same evaluations were performed to assess the IE. When an acceptable position was found, the electrode tip was heated to 90 °C for 60 s by an Inomed Neuro N50 lesion generator. Directly after coagulation, the tremor evaluations were performed once more. Until improvement in tremor was deemed satisfactory, the electrode was repositioned, and the subsequent steps were repeated.

### 2.3. Materials

**Figure 1:**
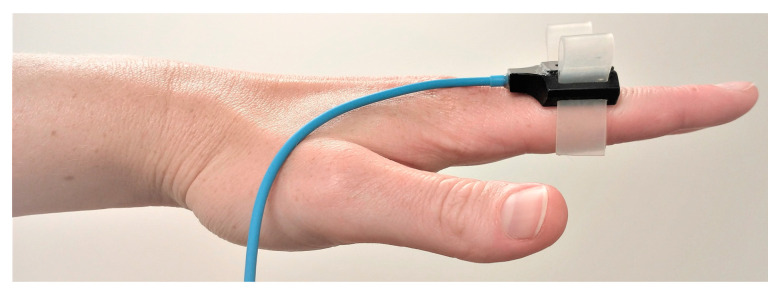
Accelerometer positioned on the left index finger.

A tri-axial accelerometer (MMA8452Q tri-Axis, Freescale Semiconductor, Inc., Austin, TX, USA) with a sampling rate of 200 Hz and range of ±2 g was used. The sensor was secured in a custom-built non-conductive plastic case and attached to the proximal phalanx of the contralateral index finger (relative to the treated hemisphere) with an adjustable silicon strap (Figure 1) [38].

Accelerometric data were recorded in LabVIEW v. 2017 (National Instruments, Austin, TX, USA) [38]. Signal analysis was performed in MATLAB v. 2022b (MathWorks, Natick, MA, USA). Statistical analysis was performed in IBM SPSS statistics v. 26 (International Business Machines Corporation, Armonk, NY, USA). MDS-UPDRS items 3.15–3.17 were used for assessment of participants [44].

### 2.4. Measurements

All measurements were performed with the participant awake and in supine position [38]. Tremor severity was assessed with three tremor tests: postural (arm stretched out), intention (finger-to-nose maneuver), and at rest. Accelerometric data were recorded during these tremor tests, while a movement disorders neurologist simultaneously assessed MDS-UPDRS scores.

**Figure 2:**
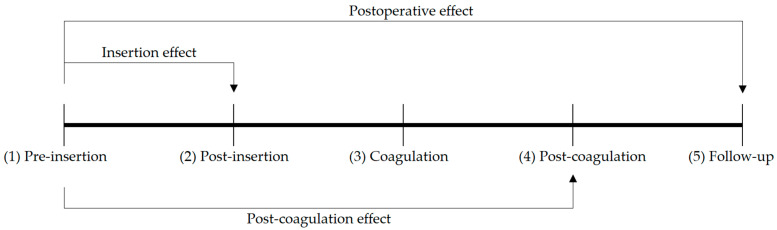
Tremor severity was measured at five time points to assess the IE, the PCE, and the PE: (1) pre-insertion and (2) post-insertion of the lesioning probe, (3) during coagulation, (4) directly after coagulation during surgery, and (5) 4–6 months follow-up.

Measurements were performed (1) before lesioning probe insertion, (2) after lesioning probe insertion, (3) during coagulation, (4) after coagulation, and (5) at 4–6 months follow-up (Figure 2). The three tremor tests were performed in steps 1, 2, 4, and 5. Steps 1 to 4 were repeated until improvement in tremor was deemed satisfactory. Accelerometric data were recorded continuously during coagulation (Figure 2).

For each outcome measure, the sum of the values of the three tremor tests at that measurement moment was used for analysis. The three different effects that were calculated for each type of summed outcome measure (SOM) were defined as follows. The insertion effect (IE) was defined as the SOM just prior to insertion of the lesioning probe (1) minus the SOM immediately post-insertion (2). The post-coagulation effect (PCE) was defined as the pre-insertion SOM (1) minus the SOM directly after coagulation (4). The postoperative effect (PE) was defined as the pre-insertion SOM (1) minus the SOM at follow-up (5), see Figure 2.

It is hypothesized that both IE and PCE correlate with PE for both MDS-UPDRS and accelerometric SOM. Second, it is hypothesized that MDS-UPDRS tremor scores correlate with accelerometric outcome measures.

### 2.5. Accelerometric Measures

Accelerometric data pre-processing was performed as described previously [38]. To assess postural, intention, and rest tremor, three outcome measures were calculated from the accelerometry data per respective test: (1) dominant frequency; (2) maximal amplitude; (3) area under the curve of power within the tremor frequency band (AUCP) [38]. The tremor frequency band was defined as 4–7 Hz for PD and ET and 2–5 Hz for HT, based on the frequencies found during preoperative recordings.

To determine the power of the acceleration norm (cm/s^2^), the periodogram power spectral density (PSD) estimate was calculated [45]. Via trapezoidal numerical integration, the AUCP in the 2–5 Hz and 4–7 Hz frequency bands was calculated from this periodogram. The dominant or peak frequency was defined as the frequency with the highest power value. For the calculation of the mean tremor amplitude, the mean of all peaks in the absolute amplitude vector was determined. This mean was multiplied by two in order to calculate the mean tremor amplitude [38].

### 2.6. Statistical Analysis

The main outcome measures used for the statistical analyses were MDS-UPDRS sum score, summed amplitude, and summed AUCP. Based on visual inspection of the histograms of the outcome variables, nonparametric tests were used for all analyses. The alpha level was determined to be 5%. Wilcoxon signed-rank test was used to analyze the IE, PCE, and PE (Figure 2). Spearman’s rank correlation coefficient (Spearman’s *ρ*) was used to calculate the strength of association between the IE and PE and between the PCE and PE. Spearman’s *ρ* was also applied to calculate the correlation between the MDS-UPDRS scores and accelerometric amplitude and between the MDS-UPDRS scores and AUCP of each tremor test.

## 3. Results

A group of 22 patients (15 men, 7 women; age (69.9 ± 11.3 years) were included. Data were gathered from 13 surgeries in 13 patients with PD, eight surgeries in seven patients with ET (one re-operation after 11 months), and three surgeries in two patients with HT (one re-operation after 14 months). In 17 out of 24 procedures, a single coagulation attempt was sufficient for a satisfactory effect on tremor severity (Table 1).

### 3.1. Quantification of Changes in Tremor during Defined Thalamotomy Stages

**Figure 3:**
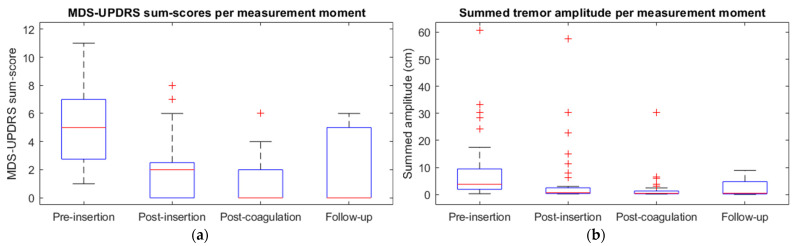
Boxplots with median and interquartile range for (**a**) MDS-UPDRS sum scores and (**b**) summed tremor amplitude at pre-insertion, post-insertion, post-coagulation, and follow-up for all participants.

Post-insertion MDS-UPDRS sum scores were significantly lower when compared to pre-insertion: 5 (1–11) vs. 2 (0–8) [*p* < 0.001]. The pre-insertion MDS-UPDRS sum scores were significantly higher than the post-coagulation sum scores, from 5 (1–11) to 0 (0–6) [*p* < 0.001]. A significant decrease was found in the postoperative MDS-UPDRS sum scores compared to the pre-insertion sum scores, from 5 (1–11) to 0 (0–6) [*p* < 0.001] (Figure 3a).

The summed tremor amplitude at pre-insertion was significantly higher when compared to post-insertion: 5 (0–61) cm vs. 1 (0–58) cm [*p* < 0.001]. The pre-insertion summed amplitude was also significantly higher than at post-coagulation, from 5 (0–61) cm to 0 (0–30) cm [*p* < 0.001]. A significant decrease was found in the postoperative summed amplitude compared to pre-insertion, from 5 (0–61) cm to 1 (0–9) cm [*p* < 0.001] (Figure 3b).

**Figure 4:**
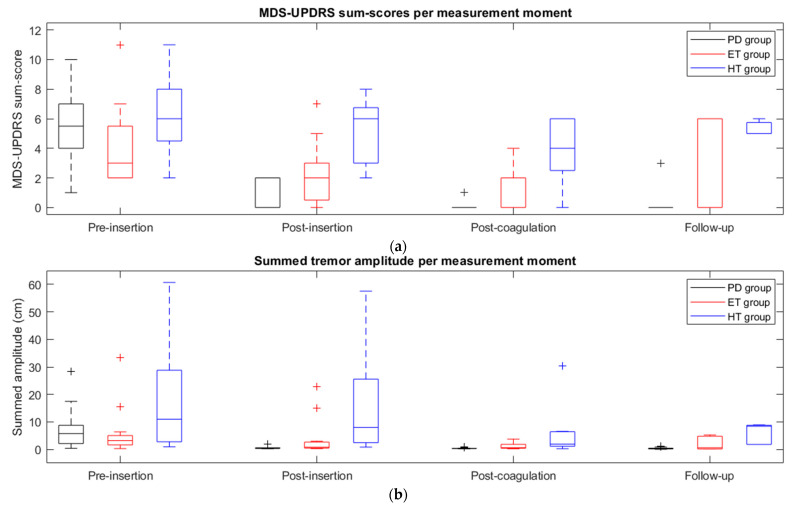
Boxplots with median and interquartile range for (**a**) MDS-UPDRS sum scores and (**b**) summed tremor amplitude at pre-insertion, post-insertion, post-coagulation, and follow-up for each group (PD group in black, ET group in red, HT group in blue).

The MDS-UPDRS sum score data per measurement moment in Figure 3a are given separately for each patient group in Figure 4a.

For the PD group, post-insertion MDS-UPDRS sum scores were significantly lower when compared to pre-insertion: 6 (1–10) vs. 0 (0–2) [*p* < 0.001]. The pre-insertion MDS-UPDRS sum scores of the PD group were significantly higher than the post-coagulation sum scores, from 6 (1–10) to 0 (0–1) [*p* < 0.001]. A significant decrease was found in the postoperative MDS-UPDRS sum scores compared to the pre-insertion sum scores of the PD group, from 6 (1–10) to 0 (0–3) [*p* < 0.001] (Figure 4a).

In the ET group, MDS-UPDRS sum scores significantly decreased from 3 (2–11) pre-insertion to 2 (0–7) post-insertion [*p* = 0.018]. The pre-insertion MDS-UPDRS sum scores of the ET group were also significantly higher than the post-coagulation sum scores, from 3 (2–11) to 0 (0–4) [*p* = 0.003]. The pre-insertion summed amplitude of the ET group decreased from 3 (2–11) to 0 (0–6) postoperatively, although this decrease was not significant [*p* = 0.387] (Figure 4a).

The post-insertion MDS-UPDRS sum scores of the HT group were lower than at pre-insertion: 6 (2–11) vs. 6 (2–8) [*p* = 0.109]. The pre-insertion MDS-UPDRS sum scores decreased significantly from 6 (2–11) to 4 (0–6) [*p* = 0.024] post-coagulation. The decrease in MDS-UPDRS sum scores at pre-insertion from 6 (2–11) to 5 (5–6) [*p* = 0.288] postoperatively was not significant (Figure 4a).

In Figure 4b, the summed tremor amplitude data per measurement moment of Figure 3b are given separately for each patient group.

The summed tremor amplitude of the PD group at pre-insertion was significantly higher when compared to post-insertion: 6 (0–28) cm vs. 0 (0–2) cm [*p* < 0.001]. The pre-insertion summed amplitude was also significantly higher than at post-coagulation, from 6 (0–28) cm to 0 (0–1) cm [*p* < 0.001]. A significant decrease was found in the postoperative summed amplitude compared to pre-insertion, from 6 (0–28) cm to 0 (0–1) cm [*p* < 0.001].

In the ET group, the summed amplitude significantly decreased from 3 (0–33) cm pre-insertion to 1 (0–23) cm post-insertion [*p* = 0.002]. The pre-insertion summed amplitude of the ET group was also significantly higher than the post-coagulation amplitude, from 3 (0–33) cm to 1 (0–4) cm [*p* = 0.002]. The pre-insertion summed amplitude of the ET group decreased from 3 (0–33) cm to 1 (0–5) cm postoperatively, although this decrease was not significant [*p* = 0.209] (Figure 4b).

The summed tremor amplitude of the HT group at pre-insertion was higher than at post-insertion: 11 (1–61) cm vs. 8 (1–58) cm [*p* = 0.075]. The post-coagulation summed amplitude of the HT group was significantly lower than that at pre-insertion: 11 (1–61) cm vs. 2 (0–30) cm [*p* = 0.018]. The decrease in summed amplitude from 11 (1–61) cm at pre-insertion to 8 (2–9) cm postoperative was not significant [*p* = 0.237] (Figure 4b).

#### During Coagulation

**Figure 5:**
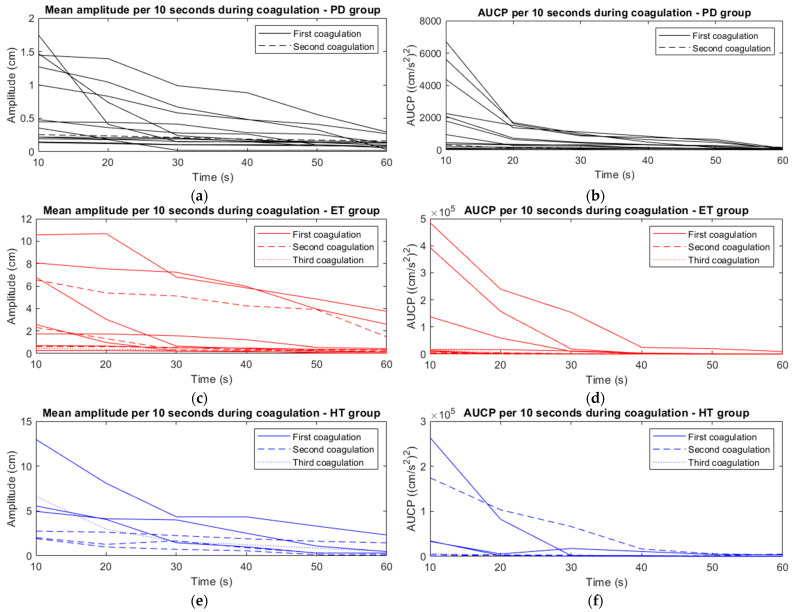
The mean amplitude per 10 s (**a**,**c**,**e**) and AUCP per 10 s (**b**,**d**,**f**) during the 60-s coagulation process, given for the PD group (**a**,**b**), ET group (**c**,**d**) and HT group (**e**,**f**).

Tremor amplitude and AUCP declined gradually during coagulation in all patients (Figure 5). The amplitudes at t = 10 s were significantly higher compared to t = 60 s, from 2 (0–13) cm to 0 (0–6) cm [*p* < 0.001]. AUCP at t = 60 s decreased significantly compared to AUCP at t = 10 s, from 5182 (cm/s^2^)^2^ (55–484,986) to 173 (cm/s^2^)^2^ (10–30,474) [*p* < 0.001]. Peak frequency did not change significantly during the coagulation process, from 3 (1–6) Hz to 3 (1–7) Hz [*p* > 0.05].

For the PD group, the amplitudes at t = 10 s were significantly higher compared to t = 60 s, from 1 (0–2) cm to 0 (0–0) cm [*p* < 0.001]. AUCP at t = 60 s decreased significantly compared to AUCP at t = 10 s in the PD group, from 705 (cm/s^2^)^2^ (55–6719) to 51 (cm/s^2^)^2^ (10–173) [*p* < 0.001].

The amplitudes in the ET group at t = 10 s were significantly higher compared to t = 60 s, from 2 (0–11) cm to 0 (0–4) cm [*p* < 0.001]. The AUCP of the ET group at t = 60 s decreased significantly compared to the AUCP at t = 10 s, from 11,382 (cm/s^2^)^2^ (329–484,986) to 295 (cm/s^2^)^2^ (101–9513) [*p* < 0.001].

For the HT group, the amplitudes at t = 10 s were significantly higher compared to t = 60 s, from 5 (2–13) cm to 0 (0–2) cm [*p* < 0.001]. AUCP at t = 60 s decreased significantly compared to AUCP at t = 10 s in the HT group, from 33,360 (cm/s^2^)^2^ (867–263,567) to 772 (cm/s^2^)^2^ (172–5301) [*p* < 0.001].

### 3.2. Relation between Intraoperative Findings and Clinical Outcome

**Figure 6:**
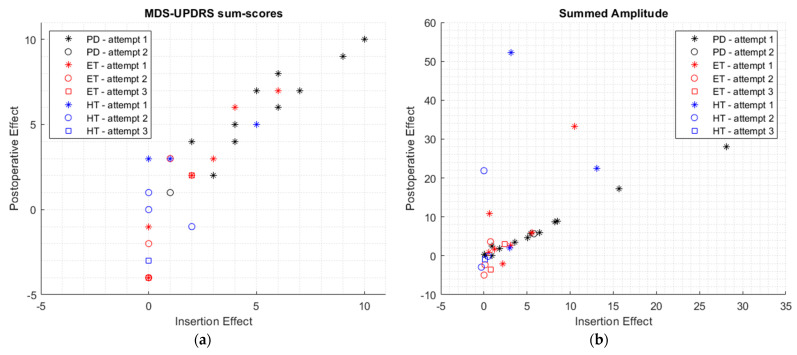
The correlation between insertion effect and postoperative effect for the MDS-UPDRS sum scores (**a**) and summed tremor amplitude (**b**), given for each group (PD group in black, ET group in red, and HT group in blue).

**Figure 7:**
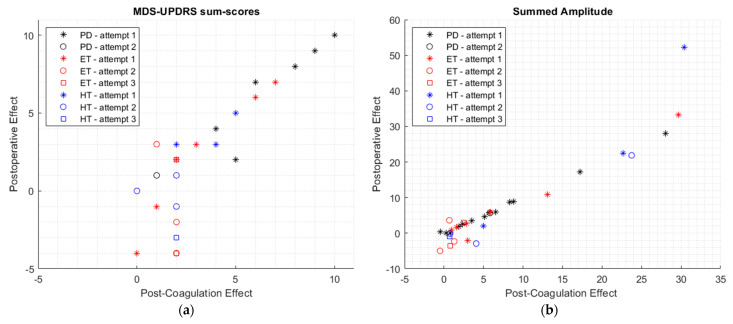
The correlation between post-coagulation effect and postoperative effect for the MDS-UPDRS sum scores (**a**) and summed tremor amplitude (**b**), given for each group (PD group in black, ET group in red, and HT group in blue).

The IE was found to be significantly associated with PE for all outcome measures [Spearman’s *ρ* > 0.690; *p* < 0.001] (Table 2). This was also the case for a correlation between the PCE and PE [Spearman’s *ρ* > 0.918; *p* < 0.001] (Table 3). The MDS-UPDRS sum scores and summed amplitude of the IE and PE were plotted per patient group and number of coagulation attempts in Figure 6. The MDS-UPDRS sum scores and summed amplitude of the PCE and PE per patient group and number of coagulation attempts are shown in Figure 7.

### 3.3. Correlation between Clinical Assessments and Accelerometric Outcome Measures

The MDS-UPDRS scores of the individual tremor tests correlated significantly with accelerometric amplitude [Spearman’s *ρ* > 0.706; *p* < 0.001]. These results were also significant for the correlation between MDS-UPDRS scores and AUCP for the individual tremor tests [Spearman’s *ρ* > 0.714; *p* < 0.001] (Table 4).

## 4. Discussion

In this study, accelerometric and clinical tremor assessments were performed during various stages of unilateral RF-thalamotomy in patients with PD, ET, and HT. Postoperative tremor intensity after unilateral thalamotomy was also studied. The aim of this research was to quantify changes in tremor intensity during surgery and to evaluate the relationship between intraoperative findings and clinical outcomes.

This study shows that tri-axial accelerometry can objectively quantify intraoperative treatment effects during RF-thalamotomy. Tri-axial accelerometry showed that tremor amplitude and AUCP declined gradually during the coagulation process and that peak frequency did not change significantly during the surgical procedure. Also, IE and PCE significantly correlated with PE at follow-up, as shown in both MDS-UPDRS ratings and accelerometric assessment. The main contribution of this study is that accelerometric measurements can provide standardized and objective input for clinical decision-making during thalamotomy. Moreover, accelerometry can be performed in real time during the coagulation process to show the change in tremor intensity.

Both tremor amplitude and AUCP declined gradually during the coagulation process, but peak frequency did not change significantly. These findings suggest that the activity of the fundamental tremor generator changes with lesioning, and probably not the generator itself. Previously, Earhart et al. reported that tremor frequency did not change with changes in Deep Brain Stimulation (DBS) of the VIM [46]. Other studies have shown that neuronal firing is inhibited during VIM-DBS [47,48], which might explain why tremor amplitude and not tremor frequency are reduced.

The IE and PCE evaluated in this study were consistent with previously reported findings [10,11,12,27,28,38,39,49]. It is generally thought that IE can predict the outcome of stereotactic surgery [27,28,50,51]. The results of this study suggest that a strong IE predicts successful tremor suppression postoperatively. Concurrently, these data indicate that the absence of both the IE and PCE predicts no change in tremor intensity after surgery. Therefore, these results suggest that the IE and PCE should be taken into account during intraoperative clinical decision-making for the replacement of the lesioning probe.

The high correlation between the IE and the PE in this study can be explained by the use of accelerometry-based outcome measures. For example, a Spearman’s *ρ* up to 0.975 was found in the AUCP outcome measure. Accelerometric measurements allow for continuous outcome measures, in contrast to discrete ordinal scales like the MDS-UPDRS. Another explanation for differences in correlation between IE and PE might be that earlier research was performed in patients undergoing DBS [27,28,50,51].

The strength of the correlations between IE and PE and between PCE and PE differed amongst the three patient groups and the number of coagulation attempts, as seen in Figure 6 and Figure 7. The relationship between IE and PE and between PCE and PE seemed to be stronger for the PD group, compared to the ET group and HT group, as seen in Figure 6 and Figure 7. No conclusions can be based on these findings as this study was not designed or powered to investigate these differences, and the small sizes of the subgroups precluded further testing of the statistical significance of these differences.

The results of this study suggest that RF-thalamotomy is an effective treatment in patients with unilateral tremors. At the same time, the medical field is evolving with alternative procedures like MRgFUS [24,25] and Gamma Knife thalamotomy [26]. These new interventions require an operative setting (e.g., MRI scanner, radioactive environment) that does not allow real-time intraoperative clinical assessment by a neurologist. Accelerometry offers a solution, providing reliable and objective measures for clinical decision-making during surgery [38,39,52]. This is supported by Baek et al., who found that accelerometry can be used for determining the location and number of MRgFUS targets to achieve optimal tremor reduction [53].

Apart from the fact that accelerometry provides rater-independent measurements, standardized accelerometric measures can settle the current uncertainty on the outcome of tremor treatment. Other benefits of this technique are that it is widely available and inexpensive (purchase costs 10 USD~30 USD), and analysis is easy and straightforward [37,38,52,54]. Also, this low-power technology does not involve disposable parts and has an estimated lifespan of at least ten years, making it a sustainable measurement method [55,56].

### Limitations

Due to the surgical setting with restricted freedom of movement for the patient, it was not possible to perform a full MDS-UPDRS assessment. Instead, only tremor items that the treatment aimed to improve were used for the measurements. Also, the results of the intraoperative measurements could have been influenced by stress, fatigue, or sedative medication effects during the surgery.

Second, all consecutive patients undergoing thalamotomy between January 2020 and June 2023 were included, even patients in whom multiple lesions were made. This might have caused discrepancies in our results, as the IE of the first attempt may have influenced the effect of the subsequent attempt. For this reason, the outcomes of the first and final attempts have been reported separately. This provides insight into how both results relate to the clinical outcome.

## 5. Conclusions

This study shows that tremor amplitude and AUCP both declined gradually during the coagulation process and that peak frequency did not change significantly during the procedures. It was also shown that both the insertion effect and the post-coagulation effect are good predictors for thalamotomy outcome. The MDS-UPDRS scores of the individual tremor tests correlated significantly with the accelerometric outcome measures. Accelerometric monitoring of tremors allows the objective quantification of these intraoperative parameters, reducing the dependency on experienced raters for reliable clinical assessments.

## Figures and Tables

**Table 1:** Demographics.

Demographic	Total Population	PD	ET	HT
Gender (percentage)	Male: 15 (68.2%) Female: 7 (31.8%)	Male: 9 (69.2%) Female: 4 (30.8%)	Male: 4 (57.1%) Female: 3 (42.9%)	Male: 2 (100.0%) Female: 0 (0.0%)
Mean (SD) age (years)	69.9 (11.3)	70.8 (8.4)	76.0 (5.2)	49.0 (10.4)
Mean (SD) disease duration (years)	8.4 (5.0)	7.9 (4.1)	11.4 (6.4)	4.4 (0.7)
Mean (SD) follow-up (months)	5.4 (1.1)	5.5 (0.9)	4.9 (1.1)	6.4 (1.2)
Treated hemisphere (percentage)	Right: 10 (45.5%) Left: 12 (54.5%)	Right: 5 (38.5%) Left: 8 (61.5%)	Right: 3 (42.9%) Left: 4 (57.1%)	Right: 2 (100.0%) Left: 0 (0.0%)
Number of coagulation attempts (SD)	1.4 (0.7)	1.1 (0.3)	1.6 (0.9)	2.3 (0.6)

**Table 2:** Spearman’s correlation between insertion effect and postoperative effect.

Coagulation Attempt	Outcome Measure	Spearman’s *ρ*	R^2^	95% CI	*p*
Only first attempts	MDS-UPDRS sum scores	0.863	0.745	0.693, 0.942	<0.001
	Summed AUCP	0.975	0.951	0.940, 0.990	<0.001
	Summed Amplitude	0.690	0.476	0.377, 0.861	<0.001
Only final attempts	MDS-UPDRS sum scores	0.967	0.934	0.920, 0.986	<0.001
	Summed AUCP	0.898	0.807	0.766, 0.957	<0.001
	Summed Amplitude	0.783	0.612	0.538, 0.906	<0.001

**Table 3:** Spearman’s correlation between post-coagulation effect and postoperative effect.

Coagulation Attempt	Outcome Measure	Spearman’s *ρ*	R^2^	95% CI	*p*
Only first attempts	MDS-UPDRS sum scores	0.950	0.903	0.882, 0.980	<0.001
	Summed AUCP	0.936	0.876	0.849, 0.973	<0.001
	Summed Amplitude	0.982	0.965	0.957, 0.993	<0.001
Only final attempts	MDS-UPDRS sum scores	0.934	0.873	0.846, 0.973	<0.001
	Summed AUCP	0.955	0.911	0.892, 0.981	<0.001
	Summed Amplitude	0.918	0.843	0.809, 0.966	<0.001

**Table 4:** Spearman’s correlation between the MDS-UPDRS scores and the accelerometric outcome measures for each type of tremor test.

Outcome Measure	Tremor Condition	Spearman’s *ρ*	R^2^	95% CI	*p*
Mean amplitude	Rest tremor	0.706	0.498	0.605, 0.784	<0.001
	Postural tremor	0.867	0.751	0.815, 0.905	<0.001
	Intention tremor	0.811	0.657	0.740, 0.863	<0.001
	All combined	0.815	0.664	0.775, 0.848	<0.001
AUCP	Rest tremor	0.714	0.510	0.616, 0.791	<0.001
	Postural tremor	0.903	0.815	0.864, 0.931	<0.001
	Intention tremor	0.731	0.535	0.638, 0.804	<0.001
	All combined	0.812	0.659	0.772, 0.845	<0.001

## Data Availability

The data may be available at a reasonable request.
